# Esophageal cancer with a double aortic arch: a case report and literature review

**DOI:** 10.1186/s13019-022-01774-1

**Published:** 2022-03-11

**Authors:** Kai Kang, Sheng Wang, Fei Xiong, Jindan Kai, Jianjian Wang, Binfeng Li

**Affiliations:** Thoracic Surgery, Hubei Cancer Hospital, Tongji Medical College, Huazhong University of Science and Technology, Hubei, 430079 China

**Keywords:** DAA, Esophageal cancer, Reconstruction route, Lymphadenectomy

## Abstract

**Background:**

Double aortic arch (DAA) is an extremely rare vascular malformation, even more so when coexisting with esophageal cancer.

**Methods:**

We report a new case of DAA with esophageal cancer recently seen at our Thoracic Tumor Clinic and review cases of DAA coexisting with esophageal cancer reported in the literature of English language from 2010 to 2020. The purposes of our literature review were to explore how to best achieve radical esophagectomy while reducing postoperative complications. The clinical manifestations, diagnostic method, surgical approach, reconstruction route, and the extent of lymphadenectomy of esophageal cancer with DAA were analyzed in detail.

**Results and conclusion:**

For such patients, 3D computed tomography is necessary for preoperative diagnosis. The surgical approach should consider factors such as the location of the tumor in the esophagus and whether the tumor is surrounded by DAA, as well as the position of the descending aorta and the requirements for the surgical field for lymphadenectomy. If esophageal reconstruction is required, the retrosternal route is preferred. We recommend that only patients with positive results of intraoperative frozen biopsy of recurrent laryngeal nerve lymph nodes should undergo three-field lymphadenectomy, which may be the best method to achieve radical esophagectomy for middle and lower esophageal cancers with DAA while minimizing postoperative complications.

**Supplementary Information:**

The online version contains supplementary material available at 10.1186/s13019-022-01774-1.

## Introduction

Double aortic arch (DAA) is a type of “vascular ring” of the aorta and a rare vascular malformation that accounts for 1–2% of congenital cardiovascular abnormalities [1]. It encircles the trachea and esophagus to form a complete vascular ring, which often causes infant respiratory symptoms and dysphagia [2, 3]. However, it is rarely diagnosed in adults because they are usually asymptomatic.

Esophageal cancer is the seventh most common cancer concerning its incidence worldwide, and the two most common histologic subtypes are squamous cell carcinoma (SCC) and adenocarcinoma (AC) [4]. DAA coexisting with esophageal cancer is extremely rare [5]. We herein report the case of a patient with DAA who underwent esophagectomy with lymphadenectomy for SCC of the thoracic esophagus and review the reports on DAA combined with esophageal cancer published in English in the past 10 years.

## Case

A 56-year-old man is presented with symptoms of dysphagia and pain in the chest and back for 3 months prior to admission. Esophagoscopy in the local hospital found an ulcerative and localized-type tumor in the esophagus between 24 and 28 cm from the incisors. Histological examination of biopsy specimens from the esophageal lesion confirmed the presence of a poorly differentiated SCC.

**Fig. 1:**
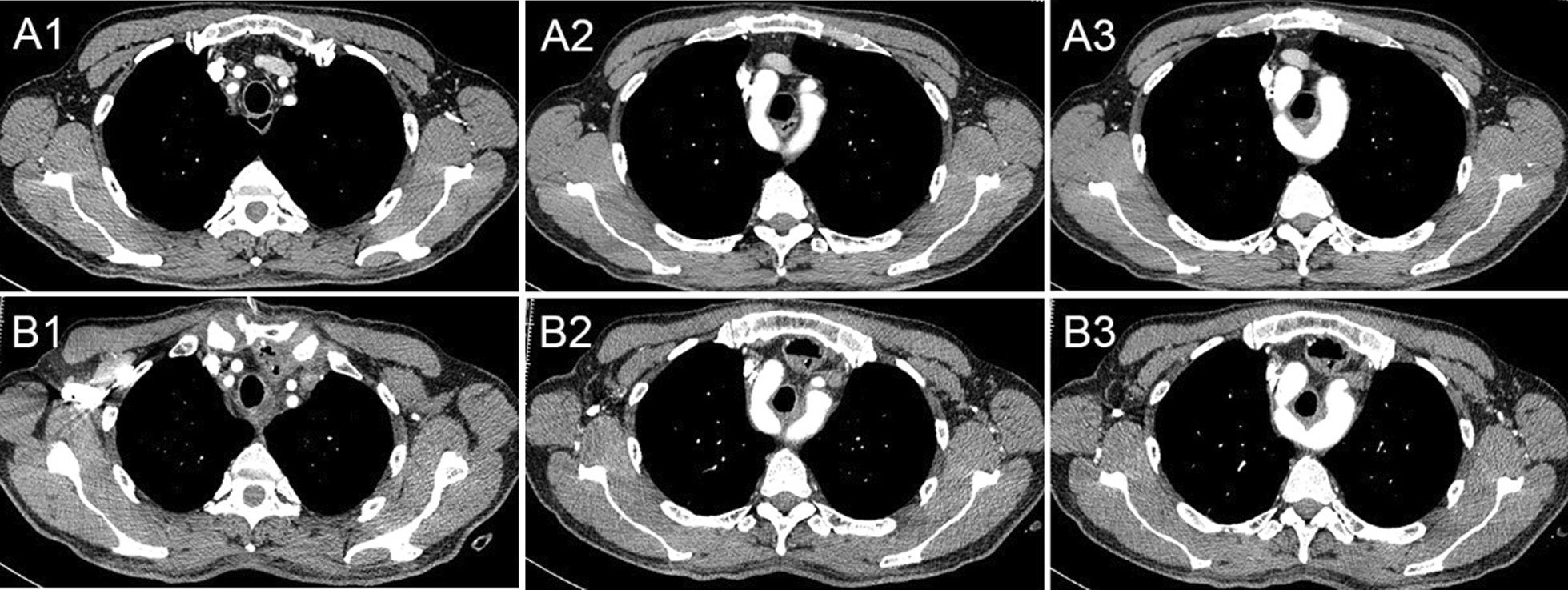
There was no brachiocephalic artery, and both the right common carotid artery and the right subclavian artery separately originated from the right aortic arch (RAA), and the left carotid arteries and subclavian arteries originated from the left aortic arch (LAA). The esophagus was displaced forward by the distal junction of both aortic arches and the descending aorta on the right side of the front of the thoracic vertebrae. The tumor was located in the esophagus between the aortic arch and the bifurcation of the trachea. CT Scans Show: Common carotid artery and subclavian artery on both sides (**A1**, preoperative;**B1**, postoperative); Vascular rings (**A2**, preoperative; **B2**, postoperative); Initial site of tumor (**A3**, preoperative; **B3**, postoperative)

**Fig. 2:**
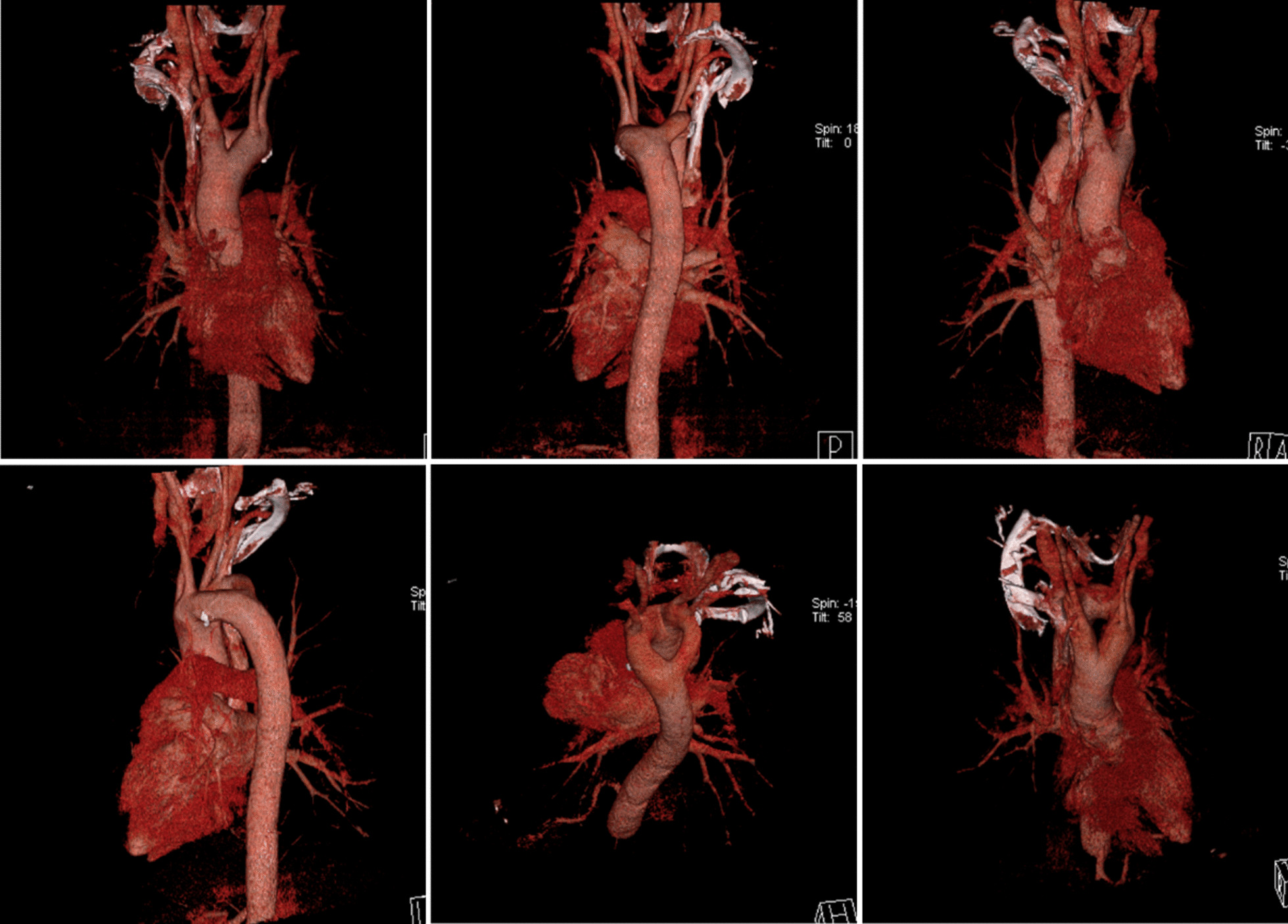
Three-dimensional computed tomography (3D-CT) Scans Show a DAA in different directions

**Fig. 3:**
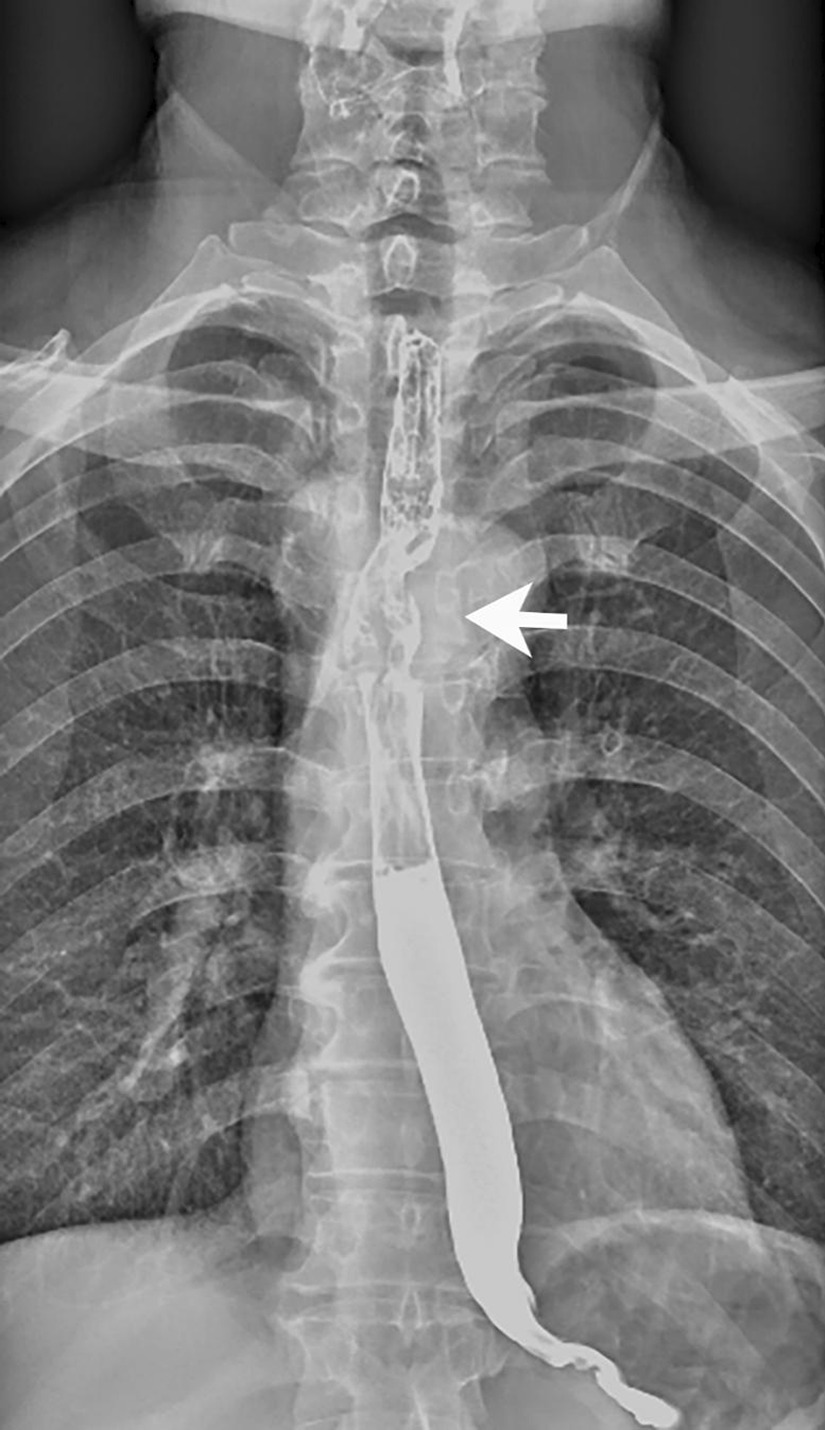
Barium esophagography revealed a filling defect

Physical examination showed no unusual findings, and laboratory investigations, including tumor markers such as SCC-related antigen and carcinoembryonic antigen, were all within normal ranges. Chest CT showed that the ascending aorta gave rise to a DAA with complete vascular rings that surrounded the main trachea and esophagus (Fig. 1). 3D-CT showed the course of the two aortic arches more clearly and comprehensively (Fig. 2). Barium esophagography revealed a 4 cm in length filling defect in the middle thoracic esophagus. The esophageal was narrow while the edges were irregular, and the mucosa was interrupted and destroyed (Fig. 3). Preoperative examination did not detect lymphadenopathy and distant metastases, so he was diagnosed as DAA with stage IIA (T3N0M0) esophageal SCC according to the TNM classification of the International Union Against Cancer Version 8 [6].

The patient underwent a subtotal esophagectomy with cervico-thoraco-abdominal three-field lymphadenectomy through a cervical inflatable mediastinoscopy combined with a right thoracoscopy, as well as esophageal reconstruction using a gastric tube through a retrosternal route, and pyloroplasty.

## Surgical procedures

**Fig. 4:**
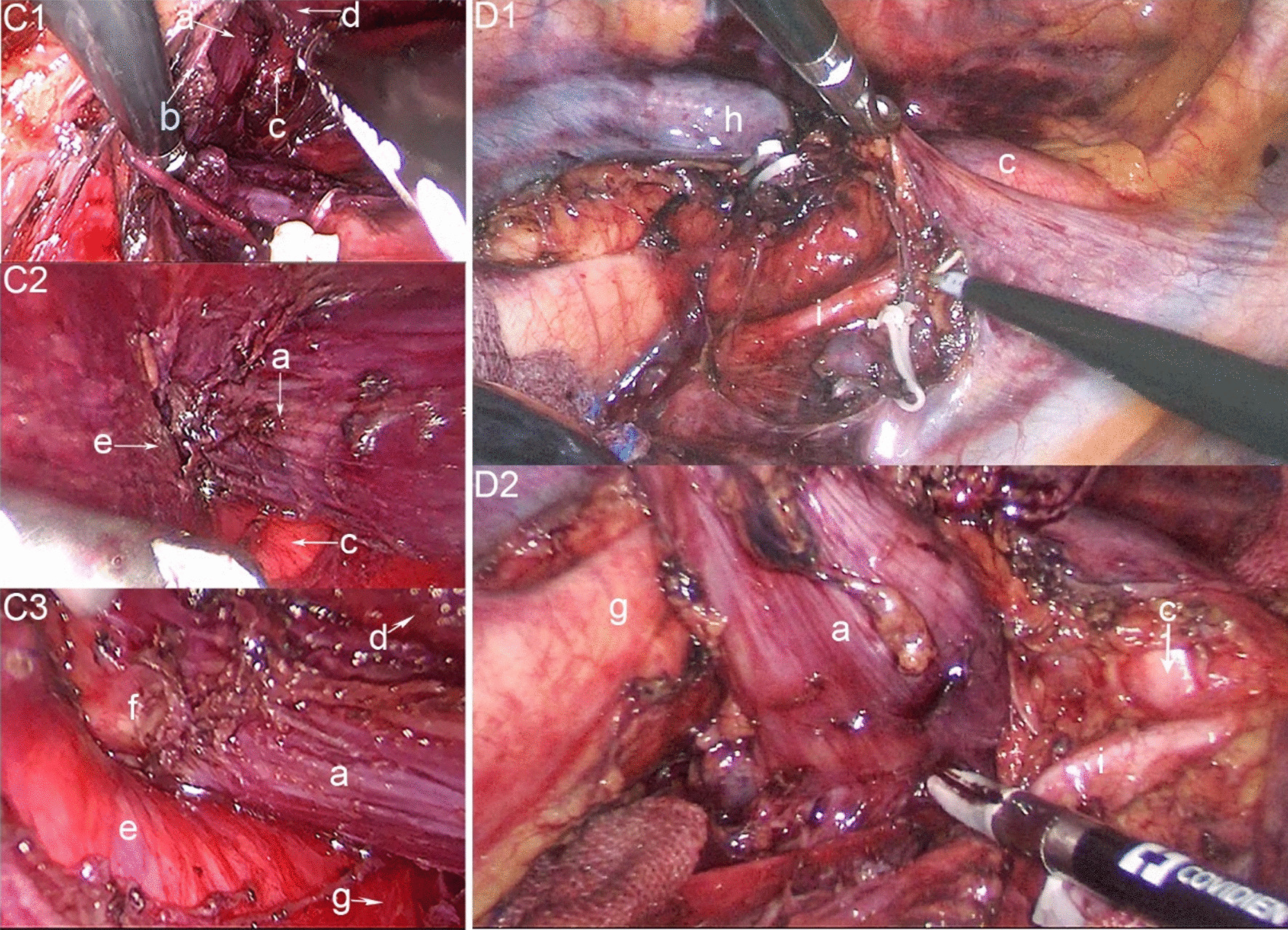
C1-3, Surgical field under Inflatable mediastinoscope; D1-2, Surgical field under right thoracoscopy. **a** esophageal, **b** left recurrent laryngeal nerve, **c** RAA, **d** trachea, **e** LAA, **f** tumor, **g** descending aorta, (h) right vagus nerve

The operation consisted of three steps. The first step was to explore the upper mediastinal using cervical aeration mediastinoscopy (Additional file 1: Video 1, Additional file 2: Video 2, Additional file 3: Video 3, Additional file 4: Video 4). A left transverse collar incision was made 1.5 cm above the clavicle, and the sternocleidomastoid muscle was mobilized to expose the sternohyoid muscle and sternothyroid muscle in sequence. The common carotid artery and internal jugular vein were exposed, and the left RLN and the esophagus were marked at the lower margin of the left thyroid. A lap-protector was inserted into the cervical wound and attached with three 5-mm trocars. The mediastinum was inflated with a positive pressure of 8–10 mmHg. We identified the left vagus nerve leading to the left RLN at the upper mediastinum (Fig. 4C). During mobilization of the esophagus via cervical mediastinoscopy, an abnormal aortic arch located on the right side of the mediastinum narrowed the area inside the vascular ring and the space between the esophagus and trachea, and superiorly the tumor was attached to the inferior pole of the vascular ring. When mediastinoscopy was performed between the DAA, we found dense fibrous tissue between the vascular ring, trachea, and esophagus. The left RLN was surrounded by fibrous tissue, and the right RLN was not found in the vascular ring. Then we anatomically exposed the left RLN, dissected the left RLN LNs and the thoracic paraoesophageal LNs.

The second step was to make a midline laparotomy incision for abdominal surgery. Mobilization of the greater curvature of the stomach was performed along the transverse colon to the spleen flexure, and the right gastroepiploic artery was preserved while the greater curvature LNs were dissected. Peritoneal trunk, left gastric artery and vein, common hepatic artery and splenic artery were separated; then left gastric vessels were ligated, and celiac artery LNs, left gastric artery LNs, right and left paracardial LNs were dissected. After dissecting the perigastric LNs, the esophageal hiatus was separated, and the abdominal esophagus was dissected. Subsequently, a gastric tube was made using a subtotal stomach and pulled up into the neck through a retrosternal route. An end-to-side anastomosis using a hand-sewn technique was performed in the left neck. In addition to routine abdominal surgical procedures, the patient was referred for pyloromyotomy and pyloroplasty.

The last step consisted of deteching the esophagus using a video-assisted right thoracic approach with the patient in the lateral recumbent position. We found the beating of the RAA in the right upper mediastinum via thoracoscopy, and the trachea and upper thoracic esophagus were framed by the DAA. To mobilize the thoracic esophagus, we ligated the azygous vein. The tumor was about 2 cm long in the middle thoracic esophagus, and the right RLN was not found along the vagus nerve at the right thoracic apex. We resected the LNs just beneath the RAA, which were defined as the right RLN LNs. The bilateral RLN LNs were sent for rapid frozen pathological biopsy. Continuing the en bloc lymphadenectomy from the Lower edge of subclavian vessels down to the esophageal hiatus, the upper thoracic paraesophageal LNs, tracheobronchial LNs, subcarinal LNs and mediastinal fatty tissues were removed carefully. The lower mediastinal lymphadenectomy was done in the usual fashion, including middle thoracic paraesophageal LNs, main bronchus LNs, lower thoracic paraesophageal LNs, pulmonary ligament LNs. Intraoperative frozen section analysis of bilateral RLN LNs showed no metastasis. The most difficult procedure of the operation was mobilizing the esophagus from the two arches and trachea, because the space between the two arches and trachea was very small. Fortunately, the location of the right RLN was not found to be the recurrent to the RAA, which is conducive to our operation procedure. We separated the esophagus from the trachea and the vessels and pulled it out through the vascular ring (Fig. 4D).

A pathological examination of the resected specimens confirmed a middle-differentiated SCC. The tumor cells invaded the entire muscle layer of the esophageal wall to the fibrous connective tissue under the muscle layer, and the nerve was found to be invaded. No metastasis was found in nodes among the 33 removed LNs. The pathological stage was pStage IIB (T3N0M0). The postoperative course was uneventful. Adjuvant therapy was not administered because the patient refused.

## Review of the literature

A research for available data was conducted in PubMed database (http://www.ncbi.nlm.nih.gov/pubmed/) using the option Advanced Search and selecting Title in the search builder and the following combinations in the search box: "double aortic arch and esophageal cancer", "double aortic arch and esophageal Carcinoma", and double aortic arch and esophagectomy. Available data as abstracts or full text articles in English and related citations and references published during the period 2010–2020 (Tables 1, 2, 3) were reviewed.

**Table 1:** The general information of 9 patients of esophageal cancer with a double aortic arch

	Age/sex	Tumor type and location	Symptoms and signs	Diagnosis method	Edward’s classification	Descending aorta
2011, Matono [5]	50y/male	SCC/Middle thoracic	Dysphasia and throat pain	CT and 3D-CT	Type IB	Right side
2012, Kubo [7]	70y/male	SCC/Middle thoracic	Dysphagia	CT and 3D-CT	Type IB	Right side
2015, Uemura [8]	63y/male	SCC/Lower thoracic	Asymptomatic	CT and 3D-CT	Type IB	Right side
2018, Peng [3]	65y/male	SCC/Middle thoracic	Dysphagia	Intraoperative found	Type IB	Right side
2019, Clement [9]	57y/male	AC/Siewert type II	Dysphagia and weight loss	CT and 3D-CT	Type IA	Right side
2019, Fujiwara [10]	64y/male	SCC/Upper thoracic	Asymptomatic	CT and 3D-CT	Type IB	Right side
2020, Mushiake [11]	79y/male	SCC/Middle thoracic	Asymptomatic	CT and 3D-CT	Type IA	Right side
2020, Kumar [12]	62y/female	SCC/Middle thoracic	Dysphagia	CT	Type IA	Right side
Our case	56y/male	SCC/Middle thoracic	Dysphagia and chest pain	CT and 3D-CT	Type IA	Right side

**Table 2:** Surgical overview of 9 patients with esophageal cancer with a double aortic arch

	Operation/approach				
	Cervical	Thoracic	Abdominal	Reconstruction	Lymphadenectomy
Matono [5]	Open	Right thoracotomy	Hand-assisted laparoscopy	Retrosternal route	Three-field
Kubo [7]	Open	Left thoracotomy	Unknown	Retrosternal route	Three-field
Uemura [8]	Open	Right thoracotomy	Unknown	Retrosternal route	Three-field
Peng [3]	No	Left thoracotomy	Unknown	Postmediastinal route	Two-field
Clement [9]	No	Left thoracosbdominal	Left thoracosbdominal	Unknown	Two-field
Fujiwara [10]	Open	Left thoracoscopy	Laparoscopy	Retrosternal route	Three-field
Mushiake [11]	Mediastinoscopy	Right thoracoscopy	Laparoscopy	Retrosternal route	Extended two-field
Kumar [12]	Open	Right thoracoscopy	Open	Right thorax route	Two-field
Our case	Inflatable mediastinoscope	Right thoracoscopy	Open	Retrosternal route	Extended two-field

**Table 3:** Summary of the past 9 case reports and our report of esophagectomy in patients with a double aortic arch

	Preoperative staging	Neoadjuvant therapy	Complication	Postoperative staging	Postoperative adjuvant therapy	Follow-up and outcome
Matono [5]	IIA(T3N0M0)	neoadjuvant chemotherapy	None	IIB(T2N1M0) (UICC 6th)	None	4 years/death
Kubo [7]	TxN0M0	No	Minor leakage	IIB(T1bN1M0) (UICC 7th)	Unknown	Unknown
Uemura [8]	TxN1M0	neoadjuvant chemotherapy	None	IIIB(T3N1M0)	Unknown	Unknown
Peng [3]	TxN0M0	No	None	IB(T1bN0M0G2)	None	Unknown
Clement [9]	TxNxM0	Neoadjuvant chemoradiation	None	IVA(T3N3M0)	Unknown	30 days/well
Fujiwara [10]	IIB(T1bN1M0)	neoadjuvant chemotherapy	Minor leakage	IA(T1bN0M0) (UICC 7th)	None	2 years/well
Mushiake [11]	I(T1bN0M0)	No	Aspiration pneumonia	I(T1bN0M0)	None	18 months/well
Kumar [12]	TxNxM0	Neoadjuvant chemoradiation	Gastric tube dilation	I(T0N0M0)	None	5 months/well
Our case	IIA (T3N0M0)	No	None	IIB (T3N0M0)	None	1 year/well

## Case reports

### Case 1

A 50-year-old man was hospitalized for dysphasia and throat pain [5]. The upper endoscopy revealed a mass in the esophagus 32–35 cm from the incisors and histological examination confirmed a moderately differentiated SCC. He was diagnosed as stage IIA (T3N0M0). CT and 3D-CT revealed a DAA. There was no brachiocephalic artery, and the bilateral carotid as well as the subclavian arteries originated respectively from each aortic arch. The patient underwent a subtotal esophagectomy with cervicothoraco-abdominal three-field lymphadenectomy through a right thoracotomy after neoadjuvant chemotherapy, and had esophageal reconstruction using a gastric tube through an retrosternal route. Pathological examination of the excised specimens confirmed a well-differentiated SCC, and one of the lesser curvature nodes (No. 3) was found to have metastasis. Therefore, the pathological stage was pStage IIB (T2N1M0). Postoperative adjuvant therapy was not administered, and he died of liver metastasis 4 years later.

### Case 2

A 70-year-old man was referred for a history of dysphagia [7]. Esophagoscopy revealed an elevated tumor 35–39 cm from the incisors, and the histological diagnosis revealed a poorly differentiated SCC. 3D-CT showed a DAA that encircled the trachea and esophagus. The bilateral carotid arteries and subclavian arteries originated from each aortic arch. The middle and lower thoracic esophagus was located on the left side of the descending aorta, and the descending aorta was on the right side of the thoracic vertebrae. The patient underwent radical esophagectomy and three-field lymphadenectomy through a left thoracotomy and esophageal reconstruction using a gastric tube through a retrosternal route. The pathological examination of the excised specimens confirmed an esophageal carcinoma, and one specimen along the right RAA as well as one at the supradiaphragm were found to have metastasis. Therefore, the pathological stage was pStage IIB (T1bN1M0).

### Case 3

A 63-year-old man was referred for an abnormality on an esophagography [8]. Histological examination of biopsy specimens confirmed that the lesion of the lower thoracic esophagus was SCC. CT and 3D-CT scans revealed the presence of a DAA with complete vascular rings surrounding the trachea and esophagus. The right carotid artery and subclavian artery originated from the RAA. The left aortic arch gave rise to the left carotid artery and subclavian artery. The esophagus was displaced forward by the distal junction of both aortic arches, and the descending aorta coursed down the right side of the lower thoracic esophagus. A PET scan revealed no distant metastases. The patient underwent esophagectomy with three-field lymphadenectomy following neoadjuvant chemotherapy and esophageal reconstruction using a gastric tube through a retrosternal route. Metastases were observed only in the perigastric nodes, and the pathological diagnosis was pT3N1M0.

### Case 4

A 65-year-old man was hospitalized for dysphagia [3]. Esophagoscopy showed an erosive surface 24–27 cm from the incisors. Histological diagnosis of the biopsy specimen confirmed the presence of an esophageal SCC. Esophagography revealed a bilateral indentation of the upper thoracic esophagus. However, CT showed the presence of an RAA, and no distant metastases were found. The patient underwent radical esophagectomy with thoraco-abdominal two-field lymphadenectomy through a left thoracotomy and esophageal reconstruction using a gastric tube through a posterior mediastinal route. A left aortic arch (LAA) was found during surgery. Postoperative 3D-CT showed a DAA surrounding the trachea and esophagus. The bilateral carotid arteries and subclavian arteries arose directly from each aortic arch. The pathological stage was IB (pT1bN0M0G2).

### Case 5

A 57-year-old man was diagnosed with a Siewert II gastroesophageal adenocarcinoma [9]. CT showed a right-sided descending aorta, and 3D-CT showed the presence of a DAA. The patient underwent radical esophagectomy with thoraco-abdominal two-field lymphadenectomy following neoadjuvant chemotherapy and radiotherapy. The proximal margin was negative on intraoperative pathology, and anastomosis was placed in the chest. There were 15 metastatic LNs diagnosed histologically, and the final pathology was ypT3N3M0. The patient was well during the 30-day postoperative visit.

### Case 6

A 64-year-old man was hospitalized for a thoracic superficial esophageal mass [10]. A histological examination of biopsy specimens confirmed the presence of SCC. CT confirmed the DAA. The right aortic arch was dominant, and the descending aorta was located at the right side of the post-mediastinum. Enhanced CT showed a metastatic LN in the right upper mediastinum. The patient was diagnosed as having cT1bN1M0 Stage IIB and a DAA. He underwent radical subtotal esophagectomy with three-field lymph node dissection following neoadjuvant chemotherapy and esophageal reconstruction using a gastric tube through a retrosternal route. The pathological diagnosis was ypT1bN0M0 Stage IA. The patient had no signs of cancer recurrence during the following 2 years.

### Case 7

A 79-year-old man was referred to after an esophageal tumor was found [11]. Histopathological examination of the biopsy samples confirmed the presence of SCC. Contrast-enhanced CT showed a DAA that encircled the trachea and thoracic esophagus. Surgery consisted of three steps. The first was dissecting the upper mediastinal LNs through cervical mediastinoscopy, and intraoperative neurophysiological monitoring (IONM) was used to monitor bilateral vagus nerves during mediastinoscopy through the cervical approach. In thoracoscopic surgery, the response on the right side can be confirmed, but the response on the left side cannot be confirmed when running IONM to stimulate both RAAs. After removing the adhesion tissue around the aortic arch, it can be confirmed that the right and left RAAs have recurred at the RAA and LAA, respectively. The last step was laparoscopic creation of a gastric tube, which was pulled to the cervical esophagus through an retrosternal route for esophagogastric anastomosis. The patient had no lymph node metastasis at any site and the pathological diagnosis was pstage I (T1bN0M0). Hedeveloped Grade II aspiration pneumonia but no hoarseness. Then, he was followed for 18 months with no signs of cancer recurrence after surgery.

### Case 8

A 62-year-old woman was hospitalized for grade 2 dysphagia [12]. She was diagnosed with locally advanced SCC of the mid-thoracic esophagus without any distant metastasis. Contrast-enhanced CT showed a DAA, which encircled the trachea and thoracic esophagus. The descending aorta was located on the right thoracic side, and the lower mid-esophagus was located on the left thoracic side. The patient underwent radical resection of esophageal cancer following neoadjuvant chemotherapy and radiotherapy. In esophageal reconstruction the gastric tube was transferred through right thorax, lateral to the right arch. The final histopathology report was ypT0N0, and the patient was asymptomatic during the 5 months of follow up.

## Discussion

1. Edwards classifications

Development of DAA occurs because of persistence of the fourth arch and dorsal aorta, leading to a complete vascular ring encircling both the trachea and esophagus and causing important respiratory and esophageal symptoms [13, 14]. The RAA is dominant in about 70% of DAA patients. In our current case, the RAA had no advantage over the LAA. The schematic presentation for malformations in the aortic arch based on embryological developmental deviations proposed by Edwards is helpful in understanding all of the possible forms of aortic arch anomalies. According to this classification [15], the patient we reported belongs to Type 1A.

2. 3D-CT is necessary in preoperative diagnostics

To safely perform radical esophagectomy in the presence of DAA, it is very important to have an accurate understanding of vessel anatomy. Spiral CT and 3D reconstruction imaging are less invasive and provide data that can be used to obtain 3D images. This method is useful for preoperative vascular assessment in patients with vascular abnormalities. A total of seven patients (including our case) have been accurately diagnosed through 3D-CT imaging preoperatively. In Peng et al.’s case [3], only RAA was found before the operation because it was not diagnosed by 3D-CT, which may not be beneficial to the surgeon. We believe that 3D-CT should be recommended for diagnosis of such patients, and accurate preoperative identification is imperative for making an operative plan.

3. Choice of surgical method

### The surgical approach should be determined according to the overall situation

At present, the main radical surgical methods for esophageal cancer include transthoracic or thoracoabdominal esophagectomy [16], which is also called Sweet esophagectomy in China [17, 18] (left thoracic, one incision), Ivor Lewis surgery [19] (right chest posterolateral and upper abdomen median, two incisions), transhiatal esophagectomy [20] (abdomen and cervical, two incisions), McKeown surgery [21] (right chest posterolateral, upper abdomen median and cervical, three incisions), and minimally invasive esophagectomy (MIE). In the future, robotic minimally invasive esophago-gastrectomy may also become one of the main surgical methods for esophageal cancer. The type of esophageal resection is decided by the tumor location, the available choices for conduit, as well as the surgeon's experience and preference altogether with the patient's preference.

In the five previously reported DAA patients with esophageal cancer, each surgical approach was different although the anatomical features of the cases were markedly similar. Matono et al. [5] chose a routine right thoracotomy. They considered that the left RLN would be found more easily through a right thoracotomy because the RAA was situated more cephalad than the LAA, which meant that the aortic window (the space beneath the aortic arch) on the right side was wider than that on the left side. Kubo et al. [7] reported a case of middle esophageal cancer with DAA. They chose to undertake a left thoracotomy because the thoracic esophagus was located on the left side of the descending aorta. They predicted that the mobilization of the esophagus and lymphadenectomy through the left thoracotomy approach would not be compromised by the presence of the descending aorta. Uemura et al. [8] did not perform the thoracotomy approach on the side opposite the descending thoracic aorta but chose the right thoracotomy approach because of a significantly swollen node in the right upper mediastinum. To help cover the left side lymphadenectomy in the lower mediastinum, they performed a transhiatal lower mediastinal lymphadenectomy. Peng et al. [3] chose a left thoracotomy for the patient because of RAA. They did not find the DAA before surgery. Clement et al. [9] reported a case diagnosed as a Siewert II gastroesophageal adenocarcinoma with DAA and a right-sided descending aorta. After careful consideration, the left thoracoabdominal approach was selected. Fujiwara et al. [10] chose the left thoracoscopic approach in a prone position because they believed that the left thoracic approach was beneficial in facilitating the visualization of the esophagus and was reasonable for a right-sided descending aorta. Moreover, left thoracoscopic surgery in the prone position has advantages in minimizing the interference of the heart in the left thoracic cavity. They reported that the heart's location in the left thorax interrupted middle to lower mediastinal dissection, in contrast to a typical right thoracic approach. Mushiake et al. [11] judged that a more familiar method is suitable for that atypical case, so they selected right thoracotomy in the prone position. Kumar et al. [12] reported that the right arch of the DAA of their patient was a nondominant arch. They believed that the absence of a dominant right arch and associated vascular anomalies will not make dissection difficult through right thorax. Therefore, they performed thoracoscopic esophageal cancer surgery through the right thorax in a semi-prone position. In addition, the patient’s tumor was below the azygous vein as well as the right arch, and the mid-lower esophagus was pushed toward the left thorax. The semi-prone position has the advantage of comfortable dissection during this part of surgery.

In our case, we chose the right thoracic approach in the lateral position because the tumor was located in the mid-thoracic esophagus. The first step was the dissection of upper mediastinal LNs and confirmation of the location of the bilateral RLN using cervical mediastinoscopy, which was similar to the method of Mushiake [11]. The inflatable mediastinoscope we use is an improvement based on Fujiwara's [22] method of using mediastinoscopy, which is different from that of Mushiake. What’s more, we peeled off the esophagus on the inner surface of the vascular ring through a mediastinoscope, which was impossible to do with a thoracic approach because the upper pole of the tumor was surrounded by DAA. We had a relatively comfortable field of vision to peel off the esophagus inside the vascular ring, which also proves that transcervical mediastinoscopy is the right choice.

### The retrosternal route should be considered more often

Esophagectomy plays a distinct role in a multimodality treatment plan for esophageal carcinoma, and the resected esophagus is most commonly reconstructed using the stomach [23, 24]. Among the multiple options for esophageal reconstruction [25, 26], two major reconstruction routes are the retrosternal route and posterior mediastinal route [27, 28]. Each reconstruction route has both advantages and disadvantages. Hu et al. [29] previously reported that the retrosternal route may be considered as a shorter way for the conduit to reach the cervical region than the posterior mediastinal route. Yang et al. [30] concluded that the posterior mediastinal route is longer than the retrosternal route and showed that both the retrosternal route and the posterior mediastinal route are safe and effective. The posterior mediastinal route was associated with shorter operation time and less blood loss compared with the retrosternal route. Due to the little available evidence, surgeons usually chose either of the route based on experience and preferences [31]. Therefore, the optimal route between posterior mediastinal and retrosternal reconstruction remains controversial, and there is a lack of clinical studies on the choice of reconstruction routes for DAA patients with esophageal cancer. Among the eight cases of esophageal cancer with DAA reported in the past, Kumar et al. [12] chose to transfer the stomach tube through right thorax, lateral to the right arch. They believed that keeping the gastric tube through right side of the posterior mediastinum would cause compression by the vascular ring and wanted to avoid additional dissection in the retrosternal route and unnecessary tension on the conduit. Peng et al. [3] chose the posterior mediastinal route, Clement et al. [9] placed the anastomosis in the left chest, and the others [5, 7, 8, 10] chose the retrosternal route. Mushiake et al. [11] believed that the posterior mediastinal route was better because it avoided the compression of the reconstruction route by the sternoclavicular joint. However, DAA patients have adhesions around the esophagus because of long-term contact between the trachea and DAA. Therefore, they finally chose the retrosternal route. We chose the retrosternal route for our patient because we believe it is the shortest one for the conduit to reach the cervical region, which can reduce tension on the esophageal-gastric anastomosis.

### Intraoperative frozen biopsy of RLN LNs is important in lymphadenectomy

There is no consensus on the optimal extent of lymphadenectomy for esophageal cancer [32–38]. On the one hand, extended lymphadenectomy has the merit of removing all potentially tumor-involved LNs and offering accurate tumor staging. On the other hand, the removal of more LNs may lead to a more invasive procedure, possibly increasing the risk of postoperative morbidity. For esophageal cancer patients with a DAA, radical surgical itself is very invasive. Therefore, it is very difficult to find a balance between ensuring sufficient LN dissection to maximize the survival rate while avoiding serious postoperative complications.

In this review, two patients with three-field lymphadenectomy and one patient with extended two-field lymphadenectomy did not have an increase in postoperative complications. Compared with patients with two-field lymphadenectomy, they did not have RLN paralysis and anastomotic leakage. One patient with three-field lymphadenectomy died of liver metastasis 4 years later. The surviving period of other patients is unknown, so it is impossible to evaluate the impact of LN dissection on the survival period. One patient was diagnosed as N1 because of the excision of the lymph node adjacent to the RLN, but it was not meaningful for there was no lack of follow-up data.

Previous studies have pointed out that RLN LN metastasis is a reliable indicator of cervical lymph node metastasis in middle/lower thoracic esophageal cancer [39–41]. Intraoperative frozen section analysis of RLN LNs is thought to be helpful in deciding whether bilateral neck dissections (three-field dissection) are indicated [41–45]. Our patient did not receive three-field lymphadenectomy since the intraoperative frozen biopsy of RLN LNs was negative. He did not receive adjuvant treatment postoperatively either, but no tumor recurrence was found in the following year.

## Conclusion

DAA coexistence with esophageal cancer is clinically rare, and the aortic arch variation may be one of the factors that promote the occurrence of esophageal cancer. Preoperative 3D-CT imaging should be performed in such patients to completely understand the complicated anomalies of the great vessels. The surgical approach should take these factors into account, such as the location of the tumor in the esophagus and whether the tumor is surrounded by DDA, as well as the position of the descending aorta in the thorax and the requirements for the surgical field for lymphadenectomy. We believe that mediastinoscopy has an advantage if the tumor is located at the DAA level and if the space is narrow between the vascular ring and the esophagus, it can provide a good surgical field for identifying the RLN in the medial annulus, which can effectively reduce the complications caused by damage to the RAAs. If gastric conduit reconstruction is required, the retrosternal route should be considered. This is the shortest path for the conduit to reach the neck area, which can reduce tension on the esophageal-gastric anastomosis. There is no consensus on the optimal extent of lymphadenectomy for esophageal cancer, but the results of intraoperative frozen biopsy of RLN LNs are helpful for choosing two-field or three-field lymphadenectomy. We recommend that only patients with positive results have three-field dissection, which may be the best method that meets the standards of radical resection of middle and lower esophageal cancer with DAA and can minimize postoperative complications.

## Supplementary Information

**Additional file 1:** The whole procedure under cervical inflatable mediastinoscopy (Part I).

**Additional file 2.** The whole procedure under cervical inflatable mediastinoscopy (Part II).

**Additional file 3.** The whole procedure under cervical inflatable mediastinoscopy (Part III).

**Additional file 4.** The whole procedure under cervical inflatable mediastinoscopy (Part IV).

## Data Availability

All data/files can be obtained from the corresponding author.

## Abbreviations

DAA: Double aortic arch
RAA: Right aortic arch
LAA: Left aortic arch
RLN: Recurrent laryngeal nerve
LNs: Lymph nodes
CT: Computed tomography
SCC: Squamous cell carcinoma
AC: Adenocarcinoma
